# Effect of Cooling Rate on Properties of Beeswax and Stearic Acid Oleogel Based on Rice Bran Oil and Sesame Oil

**DOI:** 10.3390/gels10110697

**Published:** 2024-10-27

**Authors:** Subajiny Sivakanthan, Sabrina Fawzia, Sagadevan Mundree, Terrence Madhujith, Azharul Karim

**Affiliations:** 1 School of Mechanical, Medical and Process Engineering, Faculty of Engineering, Queensland University of Technology, Brisbane, QLD 4000, Australia; ssubajiny@univ.jfn.ac.lk; 2 Department of Agricultural Chemistry, Faculty of Agriculture, University of Jaffna, Kilinochchi 44000, Sri Lanka; 3 Postgraduate Institute of Agriculture, University of Peradeniya, Peradeniya 20400, Sri Lanka; 4 School of Civil and Environmental Engineering, Faculty of Engineering, Queensland University of Technology, Brisbane, QLD 4000, Australia; sabrina.fawzia@qut.edu.au; 5 School of Agriculture and Food Sustainability, Faculty of Science, University of Queensland, St Lucia, QLD 4067, Australia; s.mundree@uq.edu.au; 6 Department of Food Science and Technology, Faculty of Agriculture, University of Peradeniya, Peradeniya 20400, Sri Lanka; tmadhujith@agri.pdn.ac.lk

**Keywords:** oil binding, oleogelation, rheology, saturated fat, structured lipid, trans fat

## Abstract

This study aimed to investigate how varying cooling rate impacts the characteristics of oleogels prepared using a sesame oil and rice bran oil blend (5:6, *w*/*w*) using a combination of beeswax and stearic acid (3:1, *w*/*w* at 12%, *w*/*w*) as the oleogelators. The study assessed three different cooling rates—0.5, 1.5, and 5 °C/min—with a focus on the attributes of the oleogels. The study revealed that the cooling rate had a substantial impact on the strength of the gel network. The cooling rate of 0.5 °C/min resulted in a higher oil-binding capacity and a stronger gel structure than fast cooling. The thermal properties and molecular interactions of the oleogels were not influenced by the cooling rate. The findings of this study indicated that the characteristics of beeswax and stearic acid oleogel prepared using sesame oil and rice bran oil blend could be tailored by manipulating the cooling rate.

## 1. Introduction

Oleogels are structured lipid systems consisting of a liquid oil phase immobilized within a network of structuring agents, such as polymers, and low molecular weight compounds. In recent years, oleogels have garnered attention as a potential alternative to solid fats, such as margarine, which are rich in trans and saturated fats. Trans fats, which are formed during the hydrogenation process, are strongly linked to health implications, including increased risk of cardiovascular diseases [1]. Solid fats containing high trans and saturated fats have traditionally been used in food products to provide stability, texture, and functionality. However, the adverse health effects linked to the excessive intake of saturated and trans fats have driven scientists to explore alternatives. Oleogels have emerged as a focal point of academic interest due to their capability to offer a trans-free and lower saturated fat option while maintaining comparable functional attributes to conventional solid fats.

It is imperative to improve oleogel characteristics in order to mimic those of conventional solid fats, ensuring the desired texture and functionality [2]. Critical factors in this endeavor include processing parameters like heating temperature, duration of heating, and cooling rate. These variables significantly influence the properties of oleogels, such as their structure, rheological behavior, stability, and functionality [3]. In the process of developing oleogels by dispersing the oleogelators directly into the oil, the oil and oleogelator mixture is heated to make a homogeneous dispersion. This is followed by cooling, which promotes microstructure formation through crystallization, thereby trapping the oil within the 3D network formed by the gelators. The process of cooling significantly influences the formation and arrangement of this microstructure, ultimately shaping the properties of the oleogel [4,5]. The elasticity, strength, and oil-holding capacity of the oleogels depend on the size, morphology, and spatial arrangement of crystals. Moreover, the cooling process exerts influence over nucleation and crystallization kinetics [5].

The correlation between cooling rate and properties of oleogels has been reported for different oleogel systems, including rice bran wax-based [6], rice bran wax, candelilla wax, and carnauba wax-based [5], as well as monoacylglycerol-based oleogels [7]. These studies have emphasized the notable influence of cooling rate on oleogel properties. This emphasizes the importance of assessing the effect of cooling rate on oleogel characteristics when establishing a new oleogel system. Even though few studies have reported on the properties of oleogels as affected by cooling rates, the correlation between cooling rate and oleogel properties has been conflicting for different oleogel systems. For instance, high cooling rates were associated with desirable oleogel properties of rice candelilla wax, bran wax, and carnauba wax-based oleogels [5], as well as monoacylglycerol oleogels [7], whereas slower cooling rates were associated with improved oleogel properties in terms of gel hardness for rice bran wax oleogel [8] and fatty alcohol-based oleogels made from peanut oil [9]. The same study by Scharfe et al. [8] reported that slow cooling resulted in softer gels in sunflower wax oleogels. In contrast to the above studies, Valoppi et al. [10] reported that the possible differences in microcrystal dimensions resulting from different cooling rates did not influence the oleogel properties. Therefore, it is worth noting that the influence of cooling rate on oleogel characteristics depends on the type of oleogelator and the resulting mechanism of formation of the oleogel [4]. Given the absence of prior research on assessing the impact of cooling rate on oleogels based on beeswax and stearic acid, it is imperative to determine the optimal cooling rate for developing an oleogel using these two oleogelators. Therefore, this study has been conducted to advance the findings of our previous study [11] to develop a healthy oleogel based on beeswax and stearic acid using a blend of sesame oil and rice bran oil. Our earlier research focused on optimizing oleogel formulations with properties similar to commercial margarines. This was accomplished using a binary mixture of sesame oil and rice bran oil, with beeswax and stearic acid as the oleogelators. We found that beeswax and stearic acid displayed synergistic effects at a 3:1 ratio. The optimized oleogel formulation consisted of 0.40 g of sesame oil, 0.48 g of rice bran oil, 0.09 g of beeswax, and 0.03 g of stearic acid (with a sesame oil to rice bran oil ratio of 5:6, and a beeswax to stearic acid ratio of 3:1, resulting in a total oleogelator concentration of 12%). This study further explored how varying cooling rates affect the oil-binding capacity, as well as the rheological, microstructural, thermal, and molecular properties of the oleogel made with a combination of beeswax and stearic acid in a sesame oil and rice bran oil blend.

## 2. Results and Discussion

### 2.1. Cooling Temperature Profile

**Figure 1:**
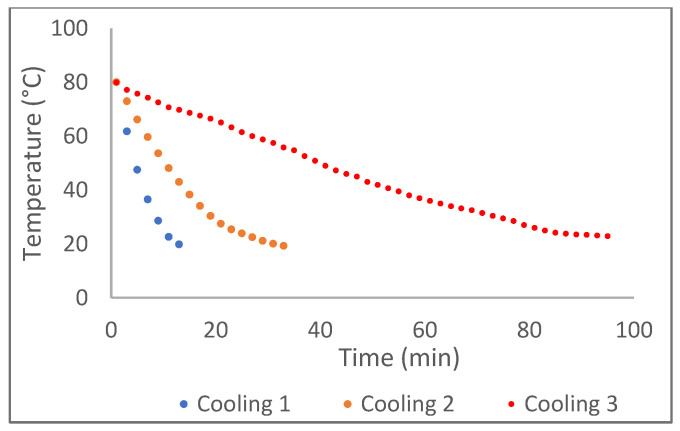
Temperature profiles recorded for the oleogels cooled at different rates. The calculated average cooling rates for cooling 1, cooling 2, and cooling 3 were 5 °C/min, 1.5 °C/min, and 0.5 °C/min, respectively.

Figure 1 illustrates the cooling temperature profiles of oleogels cooled at different rates. All samples recorded a decrease in the cooling rate with time, and the cooling rate was calculated as the average cooling rate.

### 2.2. Oil-Binding Capacity

The cooling rates were expressed as the average cooling rate, calculated by dividing the change in temperature by the time. The results demonstrated that all the samples exhibited a decline in cooling rate with time. The oil-binding capacity of the oleogels is the capability of the gel network to hold the oil, which is expressed as the fraction of oil remaining in the structure after being subjected to a centrifugal force [12]. Table 1 presents the oil-binding capacities of the oleogels. All samples had oil-binding capacities higher than 99%, indicating that more than 99% of the oil was retained within the network formed by the oleogelators after applying a centrifugal force. This exhibits the excellent oil-binding capacity of the gel structure formed by the formula evaluated. These values align with the oil-binding capacity of the same formulation, as documented in our earlier study [11]. However, a notable discrepancy was observed in the oil-binding capacities of samples prepared at varying cooling rates. The oleogel cooled at a faster rate (5 °C/min) showed significantly lower oil-binding capacity than the other two oleogels prepared by cooling at slower rates. Natural wax-based oleogels have been reported to have very high oil-binding capacity. For instance, Shi et al. [13] also demonstrated an oil-binding capacity exceeding 99% for the beeswax-based oleogel made from fish oil and sunflower oil. Even though oleogels prepared by cooling at different rates had more than 99% of the oil-binding capacity, significant differences among them indicate that there is an influence of cooling rate on the structure of the gel network. Processing conditions that influence the structure, size, and shape of the crystals, as well as their spatial distribution in the network, have an influence on the oil-binding capacity of the oleogels [14]. 

In agreement with the results of the present study, Scharfe et al. [8] also reported that rice bran wax-based gels exhibited increased hardness when cooled at slower rates, as slower cooling favored the development of more highly ordered wax crystal structures. The higher oil-binding capacity of the oleogels cooled at a slower rate in this study could be due to the fact that when an oleogel is cooled slowly, the structuring agents have more time to organize and form a well-defined three-dimensional network throughout the oil phase. It can be understood from the microstructure (Section 2.3) of the oleogels. Oleogel prepared at a faster cooling rate (5 °C/min) had smaller size crystals; however, they were less uniformly distributed compared to the other two oleogels. Blake and Marangoni [5] reported that enhancing homogeneity in crystal distribution can increase the oil-binding capacity of the oleogel. In this study, the reason for the less-ordered arrangement of the crystals at a faster cooling rate could be that there was not enough time for the organization of crystals at a faster cooling rate. Further, the impact of cooling rates on the oil-binding capacity of different oleogel systems may be different due to the differences in conformational arrangement and crystal type specific to the gelator, even though they are subjected to similar preparation methods [15]. Further explanation related to the higher oil-binding capacity at a slower cooling rate, in relation to microstructure (fractal dimension), is provided in Section 2.3.

The findings of this study regarding oil-binding capacity differ from those reported by Blake and Marangoni [5] for rice bran wax, sunflower wax, and candelilla wax-based oleogels, as well as from the results presented by Giacomozzi et al. [7] for monoglyceride oleogels. The researchers in these studies have documented that oleogels cooled at faster rates exhibit a greater oil-binding capacity compared to oleogels cooled at slower rates, and they interpreted that the smaller crystals formed by cooling at faster rates facilitate more surface area to entrap oil than those larger crystals generated at the slower cooling rates.

### 2.3. Rheological Properties

Oleogel exhibits viscoelastic behavior, displaying properties of both viscosity and elasticity. Rheological analysis of oleogels is essential for gaining a deeper understanding of the gel strength, elasticity, and viscosity of the system, which enables researchers to design the formulation and process parameters to ensure the desired functionality. In terms of rheology, oleogels can be described by storage modulus (elastic portion), G′, with an apparent plateau, and by a loss modulus, G′′. In the plateau region, G′′ is less than the G′ [16]. Analyzing the variations in G′ and G′′ with respect to frequency, as well as stress or strain, can offer valuable insights into the microstructural integrity and overall performance of oleogels across diverse applications [17].

In this study, oleogels produced by cooling at different rates were characterized by amplitude sweep, frequency sweep, and thixotropic experiment. Amplitude sweep is used to investigate how rheological properties of oleogels change with varying strain amplitudes. The region amplitude sweeps where both G′ and G′′ remain steady as strain increases is referred to as the LVR, indicating that the deformation of the structure is reversible. That is, LVR is a range of strain within which a material exhibits a linear response. After the LVR, there is a non-linear viscoelastic region where G′ and G′′ start to decrease with a rising strain [18]. During a frequency sweep, the performance of the oleogel at both low and high frequencies is assessed, revealing their behavior over the long term and short term, respectively, while maintaining a consistent strain value within the LVR. Amplitude sweep experiments were performed to evaluate the LVR and G′ at the LVR, and the oleogels were further characterized through frequency sweep and thixotropy experiments, all conducted at a consistent strain value within the LVR.

**Figure 2:**
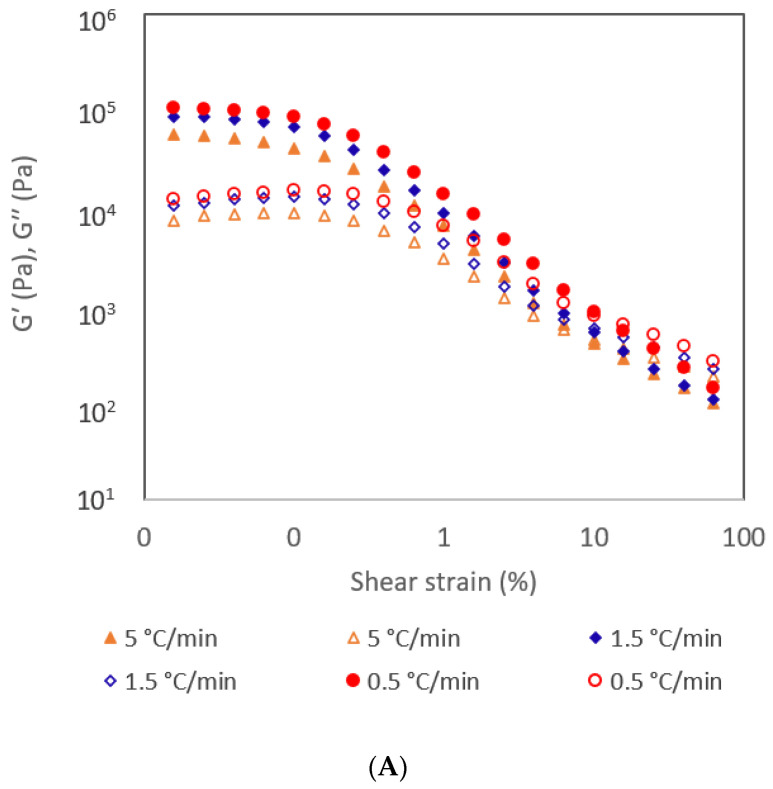

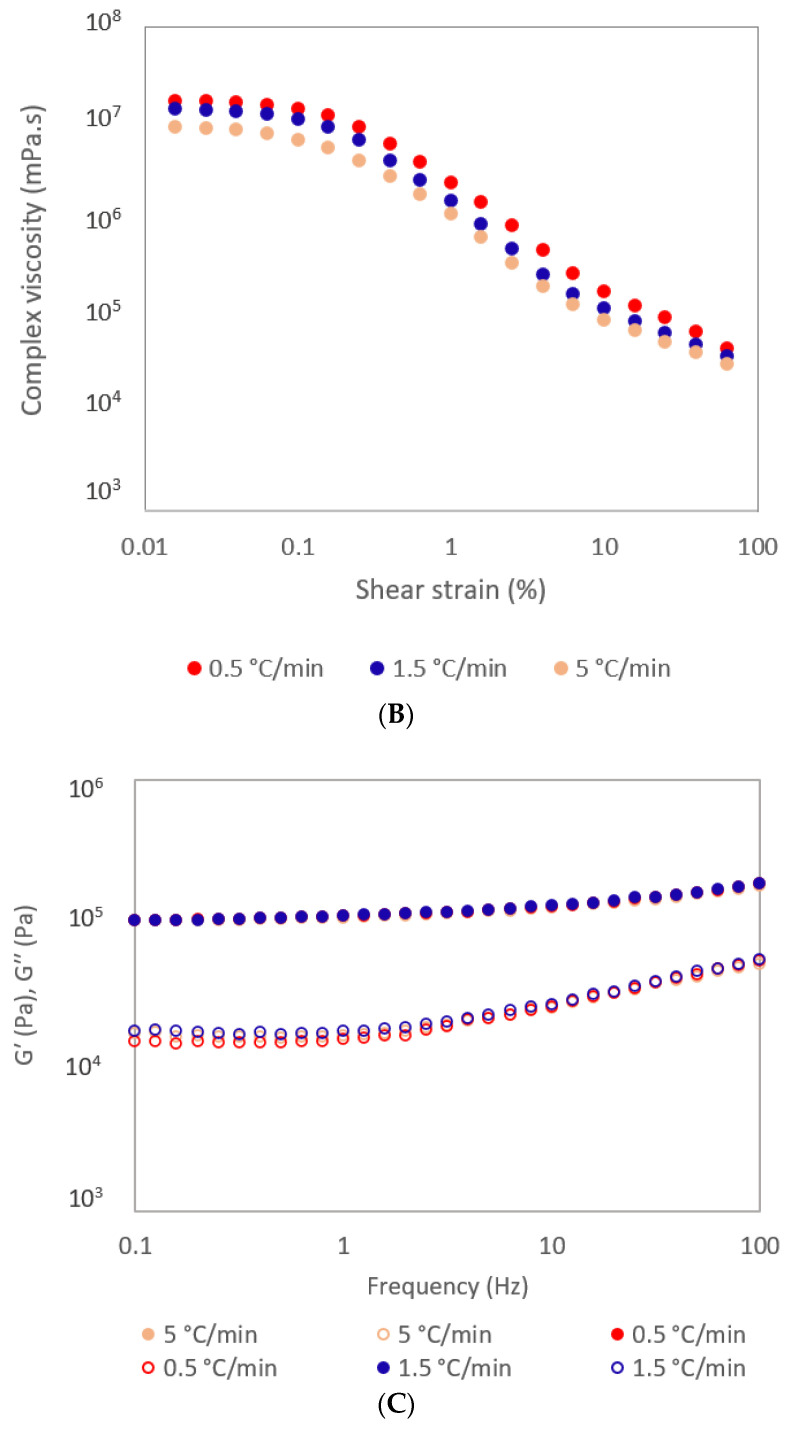

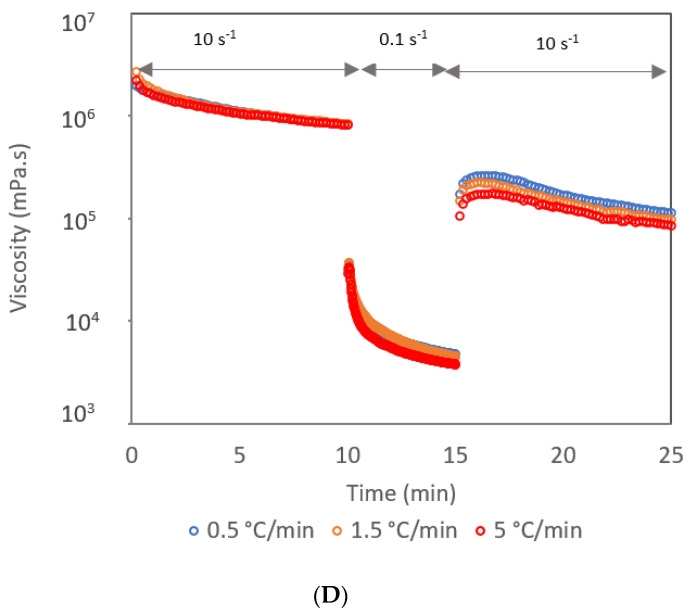
(**A**) Amplitude sweeps, (**B**) complex viscosity, (**C**) frequency sweeps, and (**D**) thixotropic behavior of oleogels produced at different cooling rates. In (**A**,**C**), solid markers represent G′ (storage modulus) and open markers represent G′′ (loss modulus).

Rheological parameters are shown in Table 1 and amplitude sweeps, complex viscosity, frequency sweeps, and thixotropic behavior are shown in Figure 2A–D. Within the LVR, it was observed that all samples displayed higher G′ values than G′′, confirming the gel-like structure of the oleogels. Specifically, the oleogel cooled at a rate of 0.5 °C/min demonstrated an elevated LVR limit and G′ at LVR in comparison to the other two oleogels that were cooled at faster rates. The plateau value of G’ within the LVR indicates the strength of the sample, and a more prolonged LVR suggests the highest resistance to structural deterioration under applied stress [19]. This is more evident from the higher structural recovery ability of the oleogels cooled at 0.5 °C/min than from the other two (Table 1 and Figure 2D). Scharfe et al. [8] have reported that cooling rates significantly altered the oleogel firmness in sunflower wax and rice bran wax oleogels; however, no significant changes were reported in the firmness of beeswax and candelilla wax oleogels. The same authors further reported that slow cooling resulted in softer gels in sunflower wax oleogels, whereas the same cooling rate resulted in harder gels for rice bran wax-based oleogels. Similarly, Trujillo-Ramírez et al. [4] also reported conflicting results for the influence of cooling rate on oleogel hardness. A faster cooling rate resulted in increased hardness of chia oil oleogels based on glycerol monostearate, whereas the same cooling rate caused a decrease in the hardness of the chia oleogel with sorbitan monostearate. Interestingly, despite these differences, both oleogels exhibited the formation of smaller crystals and a more condensed network structure. Palla et al. [20] also mentioned that a low cooling rate resulted in a greater hardness value (up to 39%) compared to fast cooling rates for the monoglyceride oleogels. Based on the existing literature and the outcomes of this study, it can be inferred that the impact of cooling rates on the mechanical strength of the oleogel depends on various factors, including the composition and type of oleogelator, and the composition and type of oil.

According to Patel et al. [21], an optimal solid fat should possess a G′ within the range of 1 × 10^5^ to 5 × 10^6^ Pa, which covers the spectrum from ‘soft’ to ‘hard’. As per the G’ in the LVR shown in Table 1, only the oleogel cooled at 0.5 °C/min had the values within this range and the other two oleogels cooled at a faster rate had G′ less than these values. That is, even though they had a gel structure, they were too soft to be considered ideal for solid fat.

The complex viscosity of the samples obtained from the amplitude sweep experiment is shown in Figure 2B. Complex viscosity is a rheological parameter that describes the resistance of a material to flow under applied stress. All samples exhibited a shear thinning (non-Newtonian) behavior; that is, the complex viscosity decreased with increasing strain. The oleogel becomes less viscous and flows more easily as the applied stress increases. With strain, the structures deform, break, or realign, leading to a decrease in the complex viscosity. Shear thinning behavior is a beneficial property of oleogels because it allows easy spreadability and flow. Shear-thinning behavior has been reported in many oleogel systems [22,23,24]. The cooling rate did not show any significant influence on the shear-thinning behavior of the oleogels.

Frequency sweeps of the samples are shown in Figure 2C. It is evident that all samples exhibited higher G′ values than G′′, confirming their solid-like behavior across all frequencies. The samples showing constant G′ with increasing frequency (frequency-independence) are categorized as strong gels, whereas samples showing increasing G′ with increasing frequency are weak gels (frequency-dependent) [17]. All samples exhibited similar behavior with increasing frequency, indicating that the cooling rate did not influence the behavior of oleogel with changing frequencies. The G′ values of all samples remained almost constant from 0.1 to 10 Hz, indicating a frequency-independent behavior, and a slight increase in the G′ afterward. Similar results were reported for different oleogel systems [19,24,25,26].

The thixotropic behavior of oleogels refers to the decrease in viscosity over time when subjected to constant stress, followed by a gradual recovery of viscosity when the stress is removed [27]. As shown in Table 1, the structural recovery percentage of the oleogel cooled at the lowest rate was significantly higher than the other two, indicating the significant influence of the cooling rate on the thixotropic properties of oleogel. Despite the same oleogel formula, the differences in the thixotropic properties of the oleogels cooled at different rates could be due to the differences in the gel structure. As indicated in the microscopic structure (below), there were prominent differences in the crystal sizes and spatial arrangements of the oleogels. The differences in the crystal arrangement could be the reason for the structural recovery ability of the oleogels. The higher oil-binding capacity, LVR, G′ at LVR, and structural recovery ability of the oleogel cooled at a slower rate indicate that slow cooling results in more desirable properties on beeswax-stearic acid oleogel compared to fast cooling rates. The studies on the effect of cooling rate on oleogel properties have not studied the influence of cooling rate on the thixotropic properties of oleogels.

### 2.4. Microstructure

**Figure 3:**
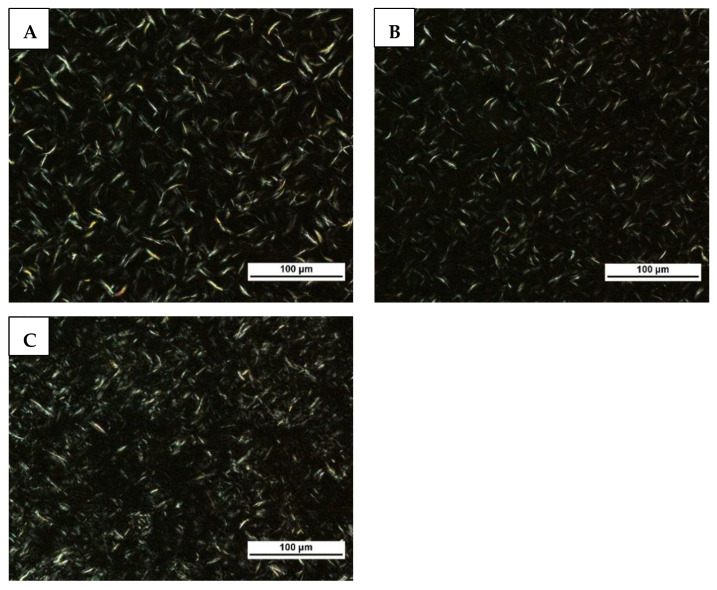
Brightfield polarized light microscopy images of oleogels cooled at 0.5 °C/min (**A**), 1.5 °C/min (**B**), and 5 °C/min (**C**). Images were acquired at 200× magnification. Scale bar 100 μm.

The microstructure of the oleogels is shown in Figure 3. Table 2 shows the fractal dimension, the length of the crystals, and the distance between the crystals in the oleogels. All samples exhibited needle-like crystals, the same as observed in our previous study with the same formula [11]. The needle-like crystal structure is a characteristic of wax-based oleogels beeswax [28]. The fractal dimension provides more detailed numerical information on the spatial arrangement of the crystals. The higher the fractal dimension, the more uniform distribution of mass with fewer cavities [29]. According to the fractal dimensions, a more uniform distribution of crystals was observed at low cooling rates than at high cooling rates. Studies have documented variations in crystal size and distribution among oleogels cooled at different rates. Notably, an increase in cooling rate resulted in the formation of smaller crystals. These findings align with previous research by Blake and Marangoni [5], Valoppi et al. [10], and Trujillo-Ramírez et al. [4]. Comparing the crystal lengths of oleogels cooled at rates of 1.5 and 5 °C/min, it is evident that both exhibited nearly identical crystal sizes, which were smaller than those observed in the oleogels cooled at 0.5 °C/min. However, the spatial distribution of crystals was more uniform in the oleogels prepared at the cooling rates of 0.5 and 1.5 °C/min compared to oleogel cooled at a faster rate. It can be understood by the distance between the crystals. The findings were consistent with those reported by Scharfe et al. [8] who also found that highly ordered wax crystal structures were formed at low cooling rates.

The reason for the poorly ordered crystals at a faster cooling rate could be that there was not enough time for the organization of crystals. In the development of sugar-based organogels, Cui et al. [30] observed that the rapid cooling process did not afford sufficient time for the nuclei of low molecular weight gelators to transition from a metastable to a stable state. The poorly ordered crystals cannot hold oil efficiently, as they may produce softer gels than highly ordered crystals. As previously discussed, the poorly ordered crystals may account for the lower strength, oil-binding capacity, and structural recovery ability of the oleogels cooled at faster rates. A higher fractal dimension increases the oil-binding capacity [20,31]. Therefore, in the present study, the higher oil-binding capacity of the oleogels cooled at a slower rate compared to the faster rate could be attributed to the higher fractal dimensions of the oleogels cooled at a slower rate.

### 2.5. FTIR Analysis

**Figure 4:**
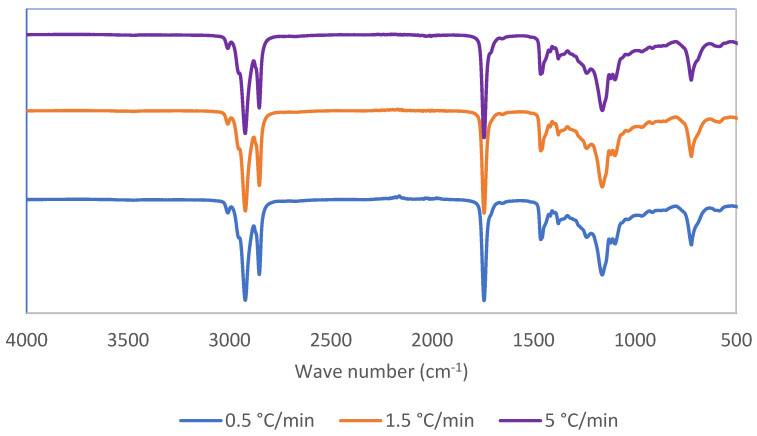
FTIR spectra of oleogels produced at different cooling rates.

FTIR spectra of the oleogels cooled at different rates are shown in Figure 4. Within the functional group region of the spectra, three distinct peaks were noted at 1744 cm^−1^ indicative of the carbonyl group, 2853 cm^−1^ representing asymmetric CH_2_ stretching, and 2923 cm^−1^ indicative of symmetric CH_2_ stretching. Notably, no noticeable spectral alterations were observed across the oleogels, suggesting that the cooling rate did not exert any notable influence on the molecular interactions among the components of the oleogel.

### 2.6. Thermal Properties

Thermal properties of oleogels, such as the onset of melting, onset of crystallization, peak melting, and peak crystallization, were evaluated using DSC. The thermal parameters are presented in Table 3. The melting and crystallization properties of oleogels cooled at different rates did not show any significant differences. This indicates that the cooling rate did not have a significant influence on the melting and crystallization properties of oleogels. Usually, melting and crystallization behaviors are determined by the oleogelators [32]. Since all oleogel samples in this study are prepared using the same formula, the thermal properties are also reported to be the same because during the DSC analysis, initially the samples were heated to 85 °C for 10 min to erase all prior crystalline history (this would have deleted all the crystal structure formed by the different cooling rates) and then the samples underwent the same heating and cooling cycles. Therefore, since the formulation was consistent, similar thermal properties were recorded for all three samples.

## 3. Conclusions

The cooling rate was found to exert a significant impact on the rheological properties, microstructure, and oil-binding capacity of the beeswax-stearic acid oleogels formulated with a blend of sesame oil and rice bran oil. The cooling rate of 0.5 °C/min yielded more desirable oleogel properties compared to fast cooling. Achieving slow cooling at room temperature presents a significant economic advantage due to its energy efficiency and ease of processing, making it a practical and cost-effective method. This study underscores that regulating the cooling rate offers a means to tailor oleogel properties. The insights gained from this research hold potential for the food industry in the manipulation and enhancement of oleogel properties, providing a viable alternative to solid fats rich in trans and saturated fats.

## 4. Materials and Methods

### 4.1. Materials

Sesame oil (Changs, Thailand) and rice bran oil (Alfa one, USA) were sourced locally in Brisbane, Australia. As analyzed using gas chromatography (GC) (Shimadzu, Japan), the fatty acid profile of the sesame oil was composed mainly of C14:0, C16:0, C18:1, and C18:2 (0.24 ± 0.03%, 9.8 ± 0.03%, 37.29 ± 0.83%, and 52.66 ± 1.21%, respectively), while the rice bran oil was composed mainly of C16:0, C18:1, and C18:2 (22.54 ± 0.40%, 43.41 ± 0.19% and 33.46 ± 0.85%, respectively). Refined beeswax, stearic acid, and chemicals and reagents were acquired from Sigma Aldrich, Australia. The melting points of beeswax and stearic acid were determined to be 62.2 ± 0.56 °C and 56.30 ± 0.14 °C, respectively, by differential scanning calorimetry.

### 4.2. Development of Oleogels

Oleogels were prepared through the direct method of oleogelation. The oleogel formulation was prepared using a blend of sesame oil and rice bran oil in a 4:5 ratio (*w*/*w*) and a mixture of beeswax and stearic acid (3:1 (*w*/*w*) ratio at a total concentration of 12% (*w*/*w*)) [11]. The mixture was heated to 80 °C while stirring continuously for 10 min using a magnetic stirrer. Afterward, the liquid mixture was cooled down to 20 °C at three different cooling rates (0.5, 1.5, and 5 °C/min) and stored for 48 h at 20 °C prior to analysis. Cooling rates such as 1.5 and 5 °C/min were achieved by keeping the sample tubes with the hot oleogel mixture at 4 °C (in a water bath) and -18 °C (in a freezer room), respectively, and the cooling rate of 0.5 °C/min was achieved by keeping the sample tubes at the ambient conditions (uncontrolled cooling). The temperature at the thermal center of the samples in identical containers (screwcap polypropylene containers with 25 mL capacity with a dimension of 100 × 65 mm (h × w)), with the same amount of sample (160 g) was measured using a thermocouple and the temperature at the center was recorded every 2 min until the temperature was reached at 20 °C. Samples were prepared in triplicates for each experiment.

### 4.3. Analytical Methods

#### 4.3.1. Oil-Binding Capacity

The oil-binding capacity of the oleogels was assessed using the method outlined by Sivakanthan et al. [11]. Briefly, 1.5 mL of the heated sample was placed into a 2 mL centrifuge tube (weight of centrifuge tube—M1) and cooled under the same oleogelation conditions at different cooling rates and incubated for 48 h at 20 °C. The weight of the tube with the gel was measured (M2) and centrifuged in a centrifuge (Eppendorf MiniSpin, Eppendorf, Leipzig, Germany) for 15 min at 13,000 rpm at 20 °C. After centrifugation, the tubes were kept inverted for 1 h to drain the liquid oil and weighed again (M3). The following formula was used to calculate the oil-binding capacity.

$$\mathrm{Oil}\;\mathrm{binding}\;\mathrm{capacity}\;(\%)=\frac{M2-M3}{M2-M1}\times 100$$

#### 4.3.2. Rheology

The oleogels were subjected to rheological analysis using a rheometer (Anton Paar MCR302 Rheometer, Graz, Austria) fitted to a Peltier temperature control system and TruStrain™ option, employing a parallel plate (sand-blasted, diameter 50 mm) maintaining an initial fixed gap of 0.5 mm. To secure a proper grip of the plate on the sample, a normal force of 0.1 N was applied. All samples were provided with a recovery time of 5 min after trimming. Triplicate analyses were performed at 20 °C. Data were analyzed using RheoCompass^TM^ version 1.30.999.

Following the procedure outlined by Aguilar-Zárate et al. [33] with minor adjustments, amplitude and frequency sweep experiments were conducted. In the amplitude sweep, a range of strain values (0.01 to 100%) was applied at a consistent frequency of 1 Hz. This allowed for the determination of the Linear Viscoelastic Range (LVR), G′ at LVR, and complex viscosity (η*). The LVR was determined as a plateau observed in both G’ and G″ from the amplitude sweeps. Subsequently, frequency sweeps were performed within the LVR, spanning from 0.01 to 100 Hz, at a fixed stress value.

The structural recovery capability of the oleogels was measured by a 3-interval thixotropy test (3ITT), Rot-Rot-Rot method. The oleogels were exposed to successive shear rates: 0.1 s^−1^ for the initial 10 min, then, 10 s^−1^ for 5 min, and then back to 0.1 s^−1^ for the final 10 min as per the methodology outlined by Patel and Dewettinck [34]. The percentage of structure recovery was calculated as described by Tavernier et al. [17].

#### 4.3.3. Thermal Analysis

Melting and crystallization characteristics of oleogels were examined using Differential Scanning Calorimetry (DSC 204 F1 Phoenix, Netzsch, Germany) as described by Sivakanthan et al. [11]. About 10 ± 1 mg of the sample was placed in aluminum crucibles, sealed and loaded into the DSC, and subjected to heating and cooling cycles under a nitrogen atmosphere. An empty crucible was used as the reference. The samples were heated to 85 °C for 10 min to eliminate any prior crystalline structure, followed by cooling to 0 °C at the rate of 2 °C/min. Then, an isothermal condition was maintained at 0 °C for 20 min, followed by reheating to 85 °C at the rate of 5 °C/min. The data were analyzed using Netzsch Proteus^®^ software (Proteus-80).

#### 4.3.4. Fourier Transform Infrared Spectroscopy (FTIR)

The FTIR spectra of the oleogels were collected using a Fourier transform infrared spectrometer (Nicolet iS50 FT-IR, Thermo Scientific, Waltham, MA, USA). Spectra were obtained by 64 scans (resolution—4 cm^−1^, range—4000 and 400 cm^−1^) at room temperature (20 °C). The OMNIC (v1.9) software was used for the analysis of peaks.

#### 4.3.5. Polarized Light Microscopy

The oleogels were cooled at three different rates (0.5, 1.5, and 5 °C/min), and their microstructures were analyzed using a polarized light microscope (Nikon Eclipse LV100ND, Nikon Instruments Inc., USA) equipped with a digital camera (Nikon DS-Fi2). A drop of hot oleogel mixture was placed onto a glass slide (preheated), covered with a coverslip, and cooled at the designated rates. The samples were then stored for 48 h at 20 °C. Then, bright-field imaging was performed at a magnification of 200× at 20 °C. Image processing was performed using ImageJ software version 1.54f (National Institutes of Health, Bethesda, MD, USA). Fractal dimension analysis was conducted using the “fractal box-counting” tool after transforming the images to 8-bit binary format with the box sizes of 2, 3, 4, 6, 8, 12, 16, 32, and 64, and a black background. To measure the distance between crystals and their lengths, the images were converted to 8-bit grayscale. Given the needle-like shape of the crystals, the distance was measured by drawing lines between the centers of the two crystals.

### 4.4. Statistical Analysis

The data were statistically analyzed using Minitab (Version 21.1) (Minitab, LLC, USA). The results are expressed as mean values with their corresponding standard deviations. To assess the significance of differences between treatments, a one-way ANOVA and Tukey’s post hoc test were applied, with a 95% confidence level (*p* < 0.05).

## Figures and Tables

**Table 1:** Oil-binding capacity and rheological parameters of oleogels produced at different cooling rates.

Average Cooling Rate	Oil-Binding Capacity (%)	Structural Recovery (%)	LVR (%)	G’ at LVR (Pa)
0.5 °C/min	99.92 ± 0.03 ^a^	35.13 ± 3.42 ^a^	0.054 ± 0.003 ^a^	107,414 ± 869 ^a^
1.5 °C/min	99.89 ± 0.04 ^a^	25.75 ± 1.43 ^b^	0.045 ± 0.001 ^b^	90,261 ± 3700 ^b^
5.0 °C/min	99.61 ± 0.02 ^b^	22.93 ± 1.87 ^b^	0.042 ± 0.001 ^b^	88,912 ± 2244 ^b^

Distinct superscript letters (a–b) within the same column indicate a significant difference (*p* < 0.05).

**Table 2:** Fractal dimension, length of the crystals, and distance between crystals in the oleogels produced at different cooling rates.

Cooling Rate	Fractal Dimension	Length of Crystals (μm)	Distance Between Crystals (μm)
0.5 °C/min	1.976 ± 0.010 ^a^	18.29 ± 4.58 ^a^	8.77 ± 4.08 ^a^
1.5 °C/min	1.567 ± 0.007 ^b^	13.42 ± 3.46 ^b^	9.02 ± 2.60 ^a^
5 °C/min	1.165 ± 0.000 ^c^	13.49 ± 2.22 ^b^	12.45 ± 8.21 ^a^

Distinct superscript letters (a–c) in the same column show a significant difference (*p* < 0.05).

**Table 3:** Thermal properties of oleogels produced at different cooling rates.

Average Cooling Rate	Onset Melting (°C)	Peak Melting (°C)	Onset Crystallization (°C)	Peak Crystallization (°C)
0.5 °C/min	26.05 ± 0.21 ^a^	49.75 ± 0.49 ^a^	48.00 ± 0.14 ^a^	47.55 ± 0.07 ^a^
1.5 °C/min	25.40 ± 0.57 ^a^	50.80 ± 0.14 ^a^	48.65 ± 0.49 ^a^	47.90 ± 0.28 ^a^
5.0 °C/min	26.35 ± 0.49 ^a^	50.25 ± 0.21 ^a^	48.65 ± 0.35 ^a^	48.15 ± 0.35 ^a^

Distinct superscript letter a in the same column shows a non-significant difference (*p* < 0.05).

## Data Availability

The data presented in this study are available on request from the corresponding author.
